# *DNAH10* mutation cause primary ciliary dyskinesia with defects of IDAf complex assembly and lung fibrosis manifestation

**DOI:** 10.1186/s13023-025-03977-w

**Published:** 2025-09-02

**Authors:** Rui Zheng, Wenhao Yang, Jierui Yan, Zhuoyao Guo, Weicheng Chen, Lina Chen, Wenming Xu

**Affiliations:** 1 Department of Obstetrics/Gynecology, Joint Laboratory of Reproductive Medicine (SCU- CUHK), Key Laboratory of Obstetric, Gynecologic, and Pediatric Diseases and Birth Defects of Ministry of Education, West China Second University Hospital, Sichuan University, Chengdu, China; 2 Department of Pediatric Pulmonology and Immunology, West China Second University Hospital, Sichuan University, Chengdu, China; 3 Key Laboratory of Birth Defects and Related Diseases of Women and Children (Sichuan University), Ministry of Education, Chengdu, China; 4 Respirology Department, Children’s Hospital of Fudan University, 201102 Shanghai, China; 5 NHC Key Laboratory of Chronobiology (Sichuan University), Chengdu, China

## Abstract

Primary ciliary dyskinesia (PCD; MIM 244400) is a genetic disorder, and its morbidity has been previously underestimated. Mutations in ciliary proteins underlie the disease, resulting in ciliary dysfunction. DNAH10 is an inner arm dynein heavy chain that has been shown to play a critical role in the movement of sperm flagella. In the present study, we demonstrated the presence of loss-of-function mutations in the *DNAH10* gene among two families affected by primary ciliary dyskinesia. Patients displayed characteristic symptoms associated with PCD, including chronic respiratory infections, productive cough, and rhinosinusitis. Additionally, a decrease in the expression of DNAH10 was confirmed in both patients. *Dnah10* knockout (KO) mice exhibited phenotypic characteristics recapitulating the PCD symptoms observed in two patients. Scanning electron microscope results showed curved and defective cilia morphology in *Dnah10* KO mice. Immunostaining also showed that DNAH10 was specifically expressed in the cilia cell. Ciliary structural studies highlighted that DNAH10 interacted with candidate PCD proteins, including CFAP57, DYNLL1, and CCDC73, contributing to the formation of a double-headed inner dynein arm f (IDAf) complex. Co-IP experiment confirmed that DNAH10 can interact with CFAP57, DYNLL1, and CCDC73. We then detected the reduced expression of CFAP57, DYNLL1, and CCDC73 in patient P02 and *Dnah10* KO mice. Furthermore, through proteomic analysis, we demonstrated alterations in the expression of abnormal innate immune proteins, super-molecular fiber organization, and mitochondrial respiratory chains. These findings suggested that the loss of DNAH10 leads to improper assembly of the IDAf complex, resulting in ciliary dysfunction and pulmonary fibrosis as the signature manifestation. Notably, our research findings hold substantial implications for the advancement of therapeutic strategies aimed at addressing ciliopathies.

**Supplementary Information:**

The online version contains supplementary material available at 10.1186/s13023-025-03977-w.

## Introduction

Primary ciliary dyskinesia (PCD, MIM 244400) is a rarer genetic disorder, whose prevalence of PCD is estimated to be approximately 1 in every 10,000 to 30,000 individuals. However, it is possible that the actual prevalence of PCD may be significantly underestimated [1]. The condition is characterized by chronic respiratory infections, abnormal positioning of internal organs, and infertility [2]. The disease arises from defects in cilia, hair-like structures lining the respiratory tract and other bodily regions [3]. Cilia play a pivotal role in clearing mucus and debris from the respiratory tract [4]facilitating fluid and particle movement in other bodily regions [5]. Given the numerous vital functions cilia perform in the body, defects in these cellular structures can lead to a diverse array of clinical manifestations. Cilia dysfunction in patients with primary ciliary dyskinesia leads to excessive mucus and debris accumulation in the respiratory tract, resulting in chronic respiratory infections such as bronchitis and pneumonia, which are often severe and recurrent [6]. Additionally, approximately 50% of individuals diagnosed with primary ciliary dyskinesia exhibit a complete reversal of their internal organs, known as situs inversus totalis [7]. Approximately 12% of individuals with primary ciliary dyskinesia exhibit heterotaxy syndrome or situs ambiguus, which is marked by malformations of the heart, liver, intestines, or spleen [8]. Infertility associated with primary ciliary dyskinesia stems from the cilia’s involvement in reproductive processes, including egg transport and sperm motility [9, 10]. Diagnosis of this condition typically involves a combination of clinical symptoms, genetic testing, and imaging techniques [11, 12].

Primary ciliary dyskinesia arises from mutations in array of genes. These genes encode instructions for synthesizing proteins that constitute the internal architecture of cilia and generate the necessary force for their bending motion [13]. Mutations in genes associated with primary ciliary dyskinesia led to cilia defects, manifesting as abnormal or immobilized ciliary movement [14]. Mutations in the *DNA11* and *DNAH5* genes constitute approximately 30% of all cases of primary ciliary dyskinesia [15]. Currently, it is known that approximately 50 gene mutations can cause PCD with recessive inheritance, but these mutations can only explain 70% PCD cases [16]. For PCD patient, stratification of patients into different classes of ciliopathic diseases has become a focus based on the recent study indicating that distinct dominant-negative disease mechanisms within the same gene underlie diverse clinical manifestations [16].

DNAH10 is a member of the dynein axonemal heavy chain (DNAH) family, encoding an inner dynein arm (IDA). To date, only one study reported one individual with primary ciliary dyskinesia carried *DNAH10* mutation [17]. Therefore, the identification of pathogenic mutations in *DNAH10* among PCD patients is crucial for confirming that *DNAH10* mutations are causative of PCD, as well as for genetic diagnosis of PCD. Besides, the precise molecular mechanism underlying the function of DNAH10 remains elusive. Future studies are warranted to elucidate the role of DNAH10 in ciliary motility and expedite drug development for PCD.

In the current study, we identified bi-allelic variants of *DNAH10* in two people with from two unrelated families by whole-exome sequencing (WES). We further generated *Dnah10* knockout (KO) mice and the *Dnah10* KO male mice resembled the phenotype observed in the two PCD patients in these families, showing chronic bronchitis, the same striking defects in cilia structure, and motile frequency. Cryo-electron microscopy uncovered DNAH10 interacted with DNAH2, DNAI3, DNAI4, DNAI7, DYNLRB1, DYNLRB2, DYNLL1, DYNLT2B, DYNLT1, CFAP43, CFAP44, CFAP57, CFAP73 CFAP100 and DNAH12 to form one double-headed inner dynein arm f (IDAf complex). DNAH10 deficiency would lead to reduced expression of DYNLL1, CCDC73, CCDC114, and CCDC39. Functionally, we observed that the absence of DNAH10 led to pathway enrichment in pulmonary fibrosis and abnormal mitochondrial metabolic processes, as revealed by proteomic analysis. Overall, our findings strongly suggested that bi-allelic variants of *DNAH10* can induce PCD in both humans and mice with fibrosis accumulation and immune dysfunction, providing significant implications for the development of therapeutic approaches targeting ciliopathies.

## Methods

### Pedigree and patient information

A 1-year-and-3-month-old girl presented with respiratory distress and dextrocardia. A 39-year-old male patient presented with recurrent respiratory infections and a productive cough, eventually seeking medical attention due to concerns about infertility. Both the two individuals and their parents were admitted for research purposes at the West China Second University Hospital. Based on exome sequencing, and clinical symptoms, the two patients were diagnosed with PCD. This study was approved by the Ethical Review Board of West China Second University Hospital, Sichuan University. Informed consent was obtained from each participant.

### Esequencing

Genomic DNA was extracted from peripheral blood samples of patients and their family members. Whole-exome sequencing (WES) was performed using the Agilent SureSelect Human All Exon V6 Kit and sequenced on the Illumina HiSeq X system. Reads were aligned to the UCSC hg38 reference genome using Burrows-Wheeler Aligner software, and PCR duplicates were removed using Picard. Variants, including non-synonymous, frameshift, splicing, start-lost, and stop-gain variations, were analyzed for pathogenicity. These variants were filtered to have frequencies less than 0.1 in public databases such as the 1 Genomes Project, gnomAD, and ExAC Browser. Missense mutations were evaluated for damaging effects using Mutation Taster and CADD. Sanger sequencing confirmed the mutations in patients and their family members, with primers listed in Supplementary Table S1.

### Pulmonary function tests

To acclimatize the mice in the cavity, they should be placed in a whole-body plethysmograph (WBP-4MR, TOW) for up to 30 min in advance, until they calmed down. Unrestrained male WT and *Dnah10* KO mice were monitored for at least 20 min, with each group comprising three mice and the experiment replicated three times (*n* = 9). WT and *Dnah10* KO mice were mixed to examine, and researcher did not know the genotype of each mouse in advance. The following parameters: maximum expiratory flow rate (PEF), time to expire 65% of the “volume” (Rt), the ratio of time to PEF to Rt (Rpef), minute volume (Mv), respiration ratio (Volbal), enhanced pause (PenH), and mid-expiratory tidal flow (EF50) values were recorde. The corresponding whole-body plethysmograph (WBP) curves were depicted by the software (ResMass 1.4.2., TOW).

### *Dnah10* KO mice information

The animal experiments were approved by the Experimental Animal Management and Ethics Committee of West China Second University Hospital, Sichuan University. All animal experiments were conducted in accordance with the regulations of the Animal Care and Use Committee of Sichuan University. The generation of *Dnah10* KO mice (C57BL/6 N) was achieved through the use of CRISPR/Cas9 technology and structural analysis, as previously described.

Oocytes were generated from super ovulated female mice (C57BL/6 N mice). Zygotes with two pronuclei were subjected to microinjection with Cas9 and sgRNA. The zygotes were then transferred into the oviducts of the pseudopregnant mice (ICR). The coat-color difference between the strain of the embryo donor (C57BL/6 N) and the pseudopregnant recipient (ICR) indicated that the newborn mice derived from the donor embryos. The founder mice and their offspring were genotyped by PCR using a Mouse Direct PCR Kit (Selleck, B40013) and Sanger sequencing analysis. A total of 21 offspring were produced from the crossbreeding of founder mice. Of these, 11 were identified as heterozygous *Dnah10* mice and were selected for the next breeding cycle. The heterozygous *Dnah10* mice and homozygous mice were used for the experiments. The expression of DNAH10 was examined using real-time PCR (RT-PCR) and western blot. The primers used for genotyping PCR are provided in Supplementary Table S1.

### Ciliary beat analysis of nasal cilia

In order to image the flow generated by nasal cilia, the nasal mucous membrane was extracted from 40-day-old *Dnah10* KO and wild type (WT) control animals and placed in a 35-mm glass-bottomed culture dish with Dulbecco’s Modified Eagle Medium/Nutrient Mixture F-12 (DMEM/F12, Gibco). The dish was placed beneath the 100× oil lens of a microscope (NIKON) and subjected to video microscopy recording at approximately 200 frames per second (fps). Subsequently, the videos were subsequently employed to quantify the ciliary beat frequency (CBF).

### Immunostaining and confocal analysis

The nasal mucous membrane from *Dnah10* KO and WT mice were digested in 2.5% trypsin (Gibco) for 10 minutes and 20 minutes, respectively. Following this, the samples were transferred to a single-cell liquid medium and terminated with fetal bovine serum (FBS). Subsequently, the samples were smeared onto slides and fixed in 4% paraformaldehyde for 30 minutes. The slides were then permeabilized with 0.5% Triton X-100, washed thrice with 1× phosphate buffered saline (PBS), and blocked with 5% bovine serum albumin (BSA) for 1 h at room temperature (RT). Then, the samples were incubated with primary antibodies at 4°C overnight. In the following days, the slides were washed thrice and incubated with secondary antibodies labelled with Alexa Fluor 488 (Thermo Fisher, 1:1000) or Alexa Fluor 594 (Thermo Fisher, 1:1000) for 2 h at RT. Finally, the slides were counterstained with 4’, 6-diamidino-2-phenylindole (DAPI) for 5 min in order to label the nuclei. Acquire the images with laser-scanning confocal microscope (Olympus).

### Hematoxylin-eosin staining (HE)

The lung and airway tissues were isolated from the WT and *Dnah10* KO mice and fixed with 4% paraformaldehyde. The tissues were then dehydrated and cleared with ethanol series (50%, 75%, 85%, 90%, 95%, and absolute alcohol) and xylene, and embedded with paraffin. Sections were made with a paraffin slicer. The paraffin sections were dewaxed and rehydrated with xylene and ethanol series. The sections were then stained with Hematoxylin for 5 min and washed for 5 min. The sections should be differentiated by 1% Hydrogen ethanol for 1 min and washed for 1 min. The sections should then be stained with 0.5% Eosin for 45 s, dehydrated and cleared with ethanol and xylene. Finally, the sections should be sealed with internal resin.

### Picrosirius red

The dewaxed and rehydrated sections, as previously stated, should be stained with Picrosirius Red for 30 min. Following this, the sections should be differentiated and dehydrated with ethanol. The sections were cleared and dehydrated with xylene. Finally, the sections were sealed with internal resin.

### Western blot

Mouse lung tissues were lysed in ice-cold RIPA buffer containing phosphatase and protease inhibitors by frozen grinding and incubated on ice for 40 min. Subsequently, sodium dodecyl sulfate (SDS) loading buffer was added into the samples, which were then heated to 95 °C for 10 min. The denatured proteins were separated using a 3 − 8% Tris-acetate gel and transferred to a 0.45-µm pore size polyvinylidene difluoride (PVDF) membrane (Millipore) at corresponding voltage. The membranes were then blocked in 5% skimmed milk for 1 h at RT and incubated with primary antibodies overnight at 4 °C. In the following day, the samples should be washed and incubated with goat anti-mouse IgG secondary antibody-HRP and goat anti-rabbit IgG secondary antibody-HRP in 5% skimmed milk at RT for 1 h. The developed immunoblot should then be visualized with ECL chemical substrate.

### Co-immunoprecipitation (Co-IP)

The 3 µg of target antibodies and 3 µg rabbit IgG were added to 200 µL of binding buffer (50 mM Tris, 150 mM NaCl, 0.3% Tween 20, pH 7.5). The antibodies mixture was then added to the Protein A/G Magnetic Beads, which had been prewashed with washing buffer (50 mM Tris, 150 mM NaCl, 0.3% Tween 20, pH 7.5) and incubated for 15 min at RT. Subsequently, the extracted proteins from mouse lung tissues were added to the antibodies-beads mixture and incubated overnight at 4 °C. Then the samples were washed thrice with washing buffer and eluted with the standard 2× SDS sample buffer and heated for 20 min at 70 °C. Finally, the co-immunoprecipitated proteins were separated using 10% sodium dodecyl sulfate-polyacrylamide gels for immunoblot analysis, as previously described.

### RT-PCR

In order to assess the efficiency of the *Dnah10*-knockdown, total RNA was isolated using the TRIzol method, which involves the use of chloroform and isopropyl alcohol. cDNAs were prepared and amplified using the Evo M-MLV RT Kit for qPCR. RT-PCR was conducted using an Applied Biosystems HT7900 with Power SYBR Green PCR Master Mix, in accordance with the manufacturer’s instructions. The primers were provided in Supplementary Table S1.

### Scanning electron microscopy (SEM) and transmission electron microscope(TEM)

For SEM, the tracheal samples from *Dnah10* KO and WT mice should be fixed in 2.5% glutaraldehyde at 4 °C overnight. The samples were washed thrice with 1× PBS and then post-fixed in 1% osmic acid for 1 h at 4 °C. Subsequently, the samples were sequentially dehydrated in an ethanol series (30%, 50%, 75%, 95%, and absolute alcohol) for 10 min. The samples were dried by a CO_2_ critical-point dryer (Eiko HCP-2) at RT. The dried specimens were then affixed to aluminum stubs, sputter-coated with an ionic sprayer meter (Eiko E-1020, Hitachi), and scanned with a field-emission SEM type Hitachi S3400.

For TEM, the samples were fixed in 3% glutaraldehyde and processed by the Chengdu Lilai Biomedicine Experiment Center following a conventional protocol. The final cilia of ultrathin tracheal epithelial cell sections were observed by TEM (TECNAI G2 F20).

### Antibody information

The antibodies used for Western blotting, Co-IP and immunofluorescence staining (IF) analyses were listed in Supplementary Table S2.

### High-speed microscopy analysis for *Dnah10* KO mouse airway organoid

To record CBF, *Dnah10* KO, WT mouse airway organoids, as well as drug-treated airway organoids were prepared at RT (25 °C). Observation of the organoids was conducted via the video microscopy with a 40× objective (Sprinter-HD Optronics). The videos were recorded at 200 fps and subsequently analyzed in a blinded manner by two researchers, as described in the Methods section. The CBF was determined using video analysis.

## Results

### Two patients diagnosed with PCD harbor bi-allelic mutations in the *DNAH10* gene

**Fig. 1:**
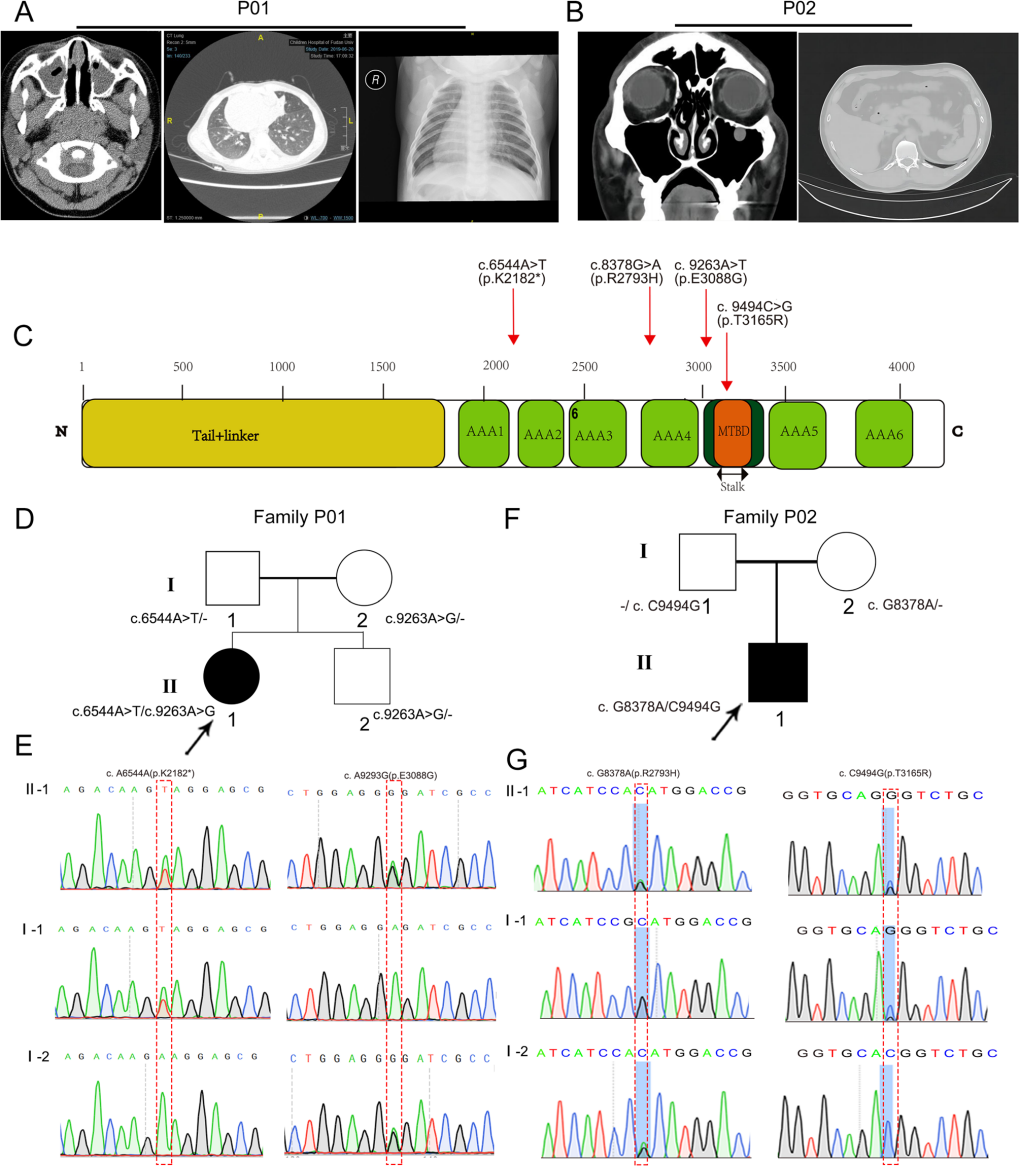
The two individuals carrying *DNAH10* mutations exhibited characteristic PCD lung pathophysiology. (**A**) Left: a nasal sinuses CT scan showing pansinusitis in the patient. Middle: a chest CT scan image revealing bronchiectasis in the patient. Right: Frontal radiography demonstrated dextrocardia (heart apex positioned on the right) in a one-year-three-month-old girl. (**B**) Left: CT scan analysis of the nasal sinuses revealed pansinusitis in the patient. Right: A chest CT scan image demonstrated the presence of small nodules. (**C**) The locations of four variants in *DNAH10*, identified in two PCD cases, were presented. The domains/motifs within DNAH10 are color-coded according to the NCBI browser. (**D**) Pedigrees of two families harboring *DNAH10* variants (P01, P02) identified through whole-exome sequencing (WES) were presented. Black-filled symbols represent two individuals with PCD within these families. Double lines signify first-degree consanguinity. (**E**) Sanger sequencing confirmed the existence of bi-allelic *DNAH10* variants in individuals II-1 of Family P01 and Family P02. The variant positions are highlighted with red boxes

A girl, 1 year and 3 months old, was born at full term via natural childbirth. She experienced respiratory distress after birth and underwent non-invasive CPAP ventilation for one week. Chest and abdominal radiographs revealed dextrocardia, lever on the left, stomach on the right. And high-resolution CT scans indicated sinusitis and patchy pulmonary infiltration, along with dextrocardia (Fig. 1A). The exhaled nitric oxide concentration through the nose was significantly reduced (patient: 13.2 nl/min, normal control of the same age: 113.4 nl/min). High-speed photography revealed that the ciliary beating in the nasal mucosa of the girl was slower compared to that of a normal individual (Supplementary Movie 1, 2). A 39-year-old male patient presented with recurrent respiratory infections, wet cough, and sought medical assistance due to infertility concerns. CT imaging revealed the presence of cystic shadows in both maxillary sinuses, while fibrosis with striated shadows and numerous small nodular shadows were observed in both lungs (Fig. 1B).

WES was performed to seek the etiology of the two individuals. Whole-exome sequencing detected *DNAH10* mutations in the girl, P01, specifically NM_207437: c.9263 A > G, p.E3088G, and c.6544 A > T, p.K2128* (Supplementary Table S3). No other pathogenic or suspected pathogenic variants that could explain the patient’s phenotype were detected. Analysis of the mutation sources from the parents revealed a compound heterozygous mutation, consistent with an autosomal recessive inheritance pattern. The c.6544 A > T mutation was derived from the mother and was a nonsense mutation, leading to premature termination of translation after amino acid 2182, resulting in a truncated polypeptide. The c.9263 A > G mutation, inherited from the father, was a missense mutation causing a change from glutamic acid to glycine at position 3088 (Fig. 1C-E). The amino acid at position 3088 is highly conserved among most species and is located within the microtubule-binding domain (MTBD). Structurally, it is difficult to directly predict the impact of this mutation, but it may affect the binding ability of DNAH10 (Fig. 1C, Supplementary Fig. 1A, B). The K2128* mutation led to the deletion of most of the DNAH10 motor domain, which may affect the assembly of the entire IDAf complex, and other proteins that directly interact with the DNAH10 motor domain (Fig. 1C, Supplementary Fig. 1A, B).

The male individual, P02, carried compound heterozygous missense mutations *DNAH10*, including c.8378G > A (p.R2793H) and c.9494 C > G (p.T3165R). The sequencing results from his parents demonstrated that the c.8378G > A and c.9494 C > G mutations were inherited from his father and mother, respectively (Fig. 1F, G). The amino acid at position 2793 and 3165 are highly conserved among most species and are located within the Hydrolytic ATP binding site of dynein motor region D4 and MTBD, respectively (Fig. 1C, Supplementary Fig. 1A).

All the identified *DNAH10* variants were either rare or absent in public human databases, including the 1000 Genomes Project and gnomAD (Supplementary Table S3). Additionally, these missense variants in *DNAH10* were predicted to be damaging using tools such as SIFT, MutationTaster, Proven prediction (Supplementary Table S3).

### Impact of biallelic variants on DNAH10 expression

**Fig. 2:**
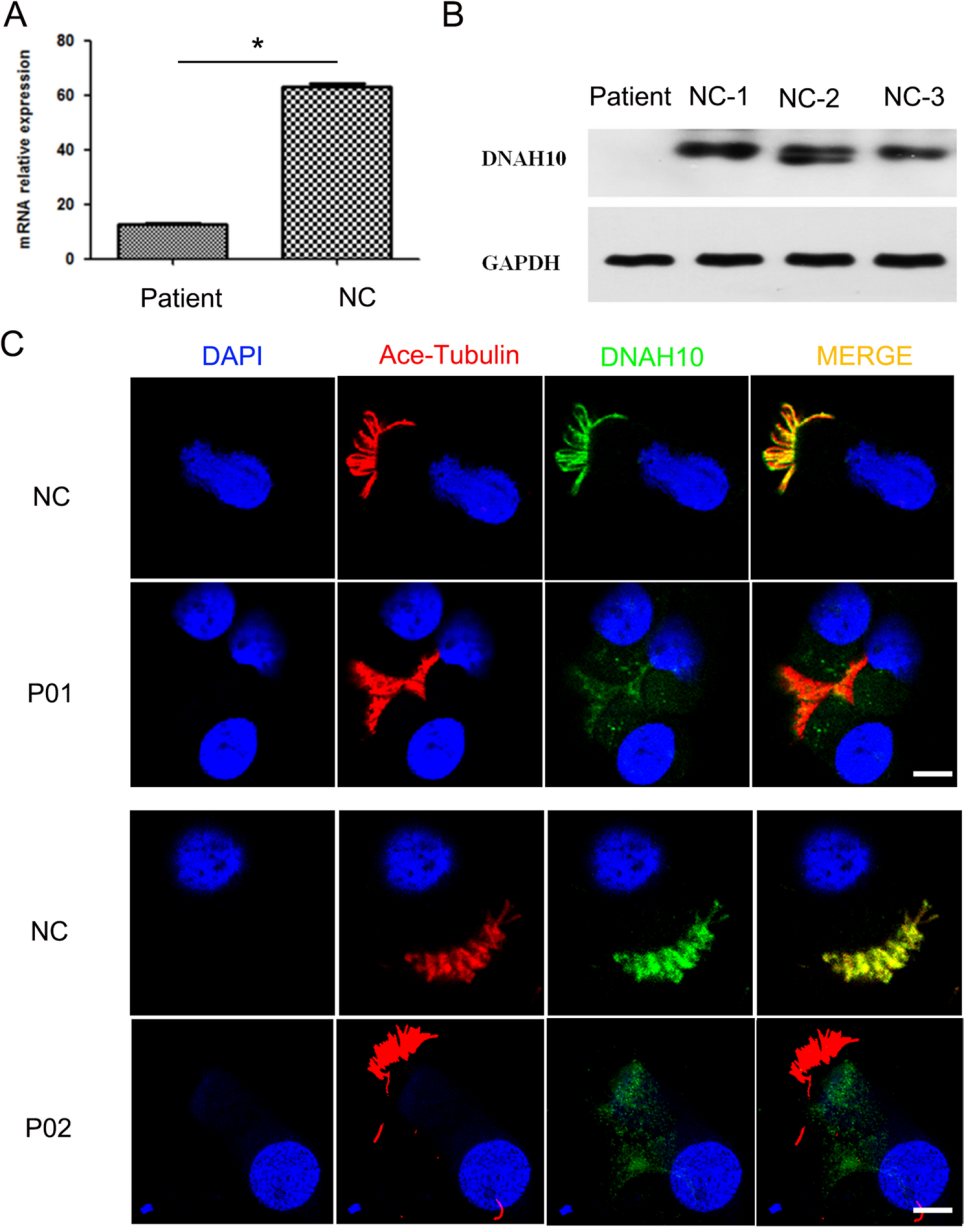
Bi-allelic *DNAH10* variants induced declined expression of DNAH10. (**A**) RT-PCR was employed to evaluate the expression of *DNAH10* at the RNA level in nasal mucosal tissue collected from P01. *P < 0.05. *n* = 3 per group (**B**) Western blot analysis was conducted to assess the expression of DNAH10 in nasal mucosal tissue. (**C**) Representative images were obtained from the nasal mucosa of control individuals (NC) and individuals harboring bi-allelic *DNAH10* variants. These images were stained with the anti-DNAH10 antibody, anti-α-tubulin antibody, and DAPI. Scale bars = 5 mm. Red, Ace-tubulin; Green, DNAH10, respectively

To assess the in vivo effects of these variants on *DNAH10*, we initially employed RT-PCR to examine the transcriptional expression levels of *DNAH10* in ciliated epithelial cells of nasal mucosa. The results indicated a significant decrease in DNAH10 expression in the patient P01 (Fig. 2A). Western blot analysis demonstrated nearly absent DNAH10 protein expression in ciliated epithelial cells of patient P01, in contrast to those without PCD (Fig. 2B). Subsequently, we conducted immunofluorescence analysis to investigate alterations in DNAH10 expression and localization. Notably, in contrast to the nasal mucosa cilia of healthy individuals, where distinct DNAH10 staining was observed along the ciliary axoneme, no DNAH10 staining was detected in the nasal mucosa cilia of the two subjects (Fig. 2C). This finding suggested that the biallelic variants may lead to a decline in DNAH10 expression.

### *Dnah10* KO mice display typical PCD phenotypes consistent with IDA defects

To investigate the impact of DNAH10 deletion on cilium function in vivo, we first developed a CRISPR-Cas9-based *Dnah10* KO mouse model (Supplementary Figure S2A). The genotypes of the mice were verified through Sanger sequencing and PCR analysis of genomic tail DNA (Supplementary Figure S2B, C). Western blot analysis further revealed that DNAH10 protein was largely undetectable in KO mice (Supplementary Figure S2D). Additionally, RT-PCR analysis demonstrated the absence of the *Dnah10* transcript in the lung tissues of KO mice (Supplementary Figure S2E). Furthermore, immunofluorescence staining revealed a significant reduction in ciliary DNAH10 expression in ciliated cells of *Dnah10* KO mice lung tissues (Supplementary Figure S3).

**Fig. 3:**
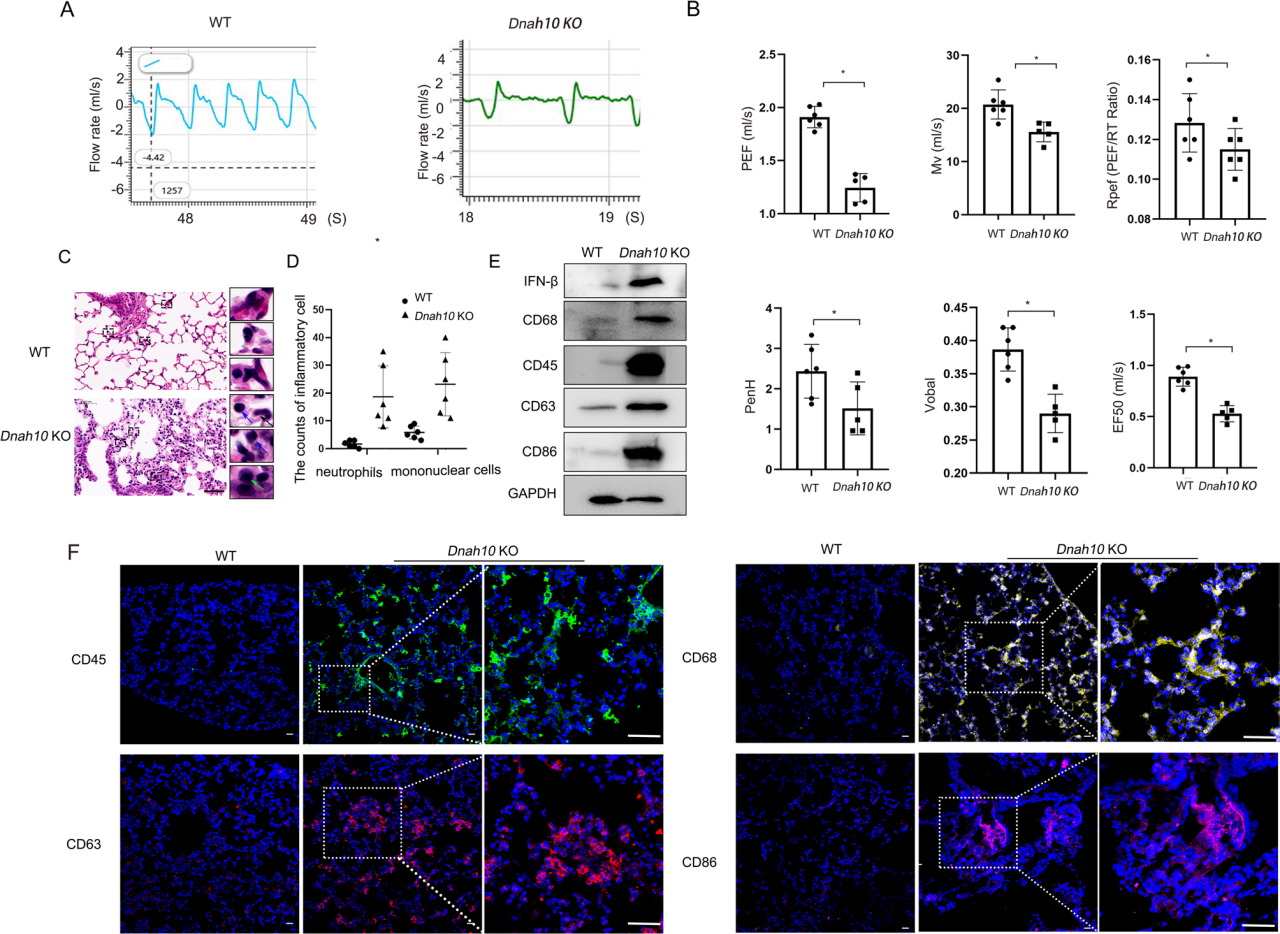
*Dnah10* KO mice represented compromised lung function and accumulated inflammation. (**A**) Breathing curve illustrating lung function parameters in WT and *Dnah10* KO mice. (**B**) Lung function parameters indicate airway obstruction and chronic bronchitis in *Dnah10* KO mice. PEF: peak expiratory flow rate (mL/s); Mv: minute ventilation (mL); Rpef: ratio of time to PEF to Rt, reflecting respiratory muscle strength and small airway obstruction; Rt: time to expire 65% of tidal volume; Volbal: respiration ratio; Penh: measure of bronchoconstriction; EF50: expiratory flow at 50% exhalation (mL/s), indicating obstructive ventilatory dysfunction. *P < 0.05; *n* = 6/group. (**C**) H&E staining of lung sections from 40-day-old WT and *Dnah10* KO mice revealed increased infiltration of inflammatory cells in *Dnah10* KO mice compared to WT mice. Mononuclear cells, including lymphocytes and macrophages, as well as neutrophils, were significantly elevated, as illustrated in the scatter plot. The black dotted boxes highlight the magnified areas of the lung sections. Lymphocytes, neutrophils, and macrophages were indicated by green, black, and blue arrows, respectively. Scale bar = 20 μm. (**D**) Cell counting for neutrophils and mononuclear cells from left HE staining. Equal-sized 5% areas of the sections were selected. The statistical analysis was presented in the accompanying panel. *P < 0.05; *n* = 6/group. (**E**) Western blot analysis was conducted to evaluate the expression levels of inflammatory factors, including IFN-β, CD68, CD45, CD63, and CD86, in *Dnah10* KO and WT mice. (**F**) Immunostaining of immunocytes, including CD45 (leucocyte marker), CD68 (macrophage marker), CD86 (marker for monocytes, T, and B lymphocytes), and CD63 (marker for activated basophils), revealed enhanced positive signals in the lung sections of the *Dnah10* KO mouse model. Green, CD45; yellow, CD68; red, CD63; purple, CD86; blue, DAPI. Scale bar = 50 μm

Mouse pulmonary function was determined by following parameters, including inspiratory-to-expiratory time (Ti/Te), PEF, Mv, Rpef, Volbal, PenH, and EF50. The results showed that the above parameters were significantly reduced in the *Dnah10* KO mice, indicating increasing airway obstruction and worsening bronchoconstriction (Fig. 3A, B). Hematoxylin and eosin (H&E) staining demonstrated chronic lung infection, which induced inflammatory cell infiltrates and pulmonary interstitial hyperplasia (Fig. 3C). An increased number of neutrophils and mononuclear cells was observed, including lymphocytes and macrophages, in the lung of *Dnah10* KO mice (Fig. 3D). Western blot analysis demonstrated significantly elevated levels of inflammatory cytokine IFN-β and complement regulatory molecule CD46, accompanied by increased expression of immune cell-specific markers, including CD68 (monocyte), CD45 (T cell), CD63 (neutrophil), and CD86 (dendritic cell) (Fig. 3E). The fluorescence intensity of these inflammatory cell markers was significantly intensified in the lungs of *Dnah10* KO mice, showing severe respiratory tract infections in these animals (Fig. 3F).

**Fig. 4:**
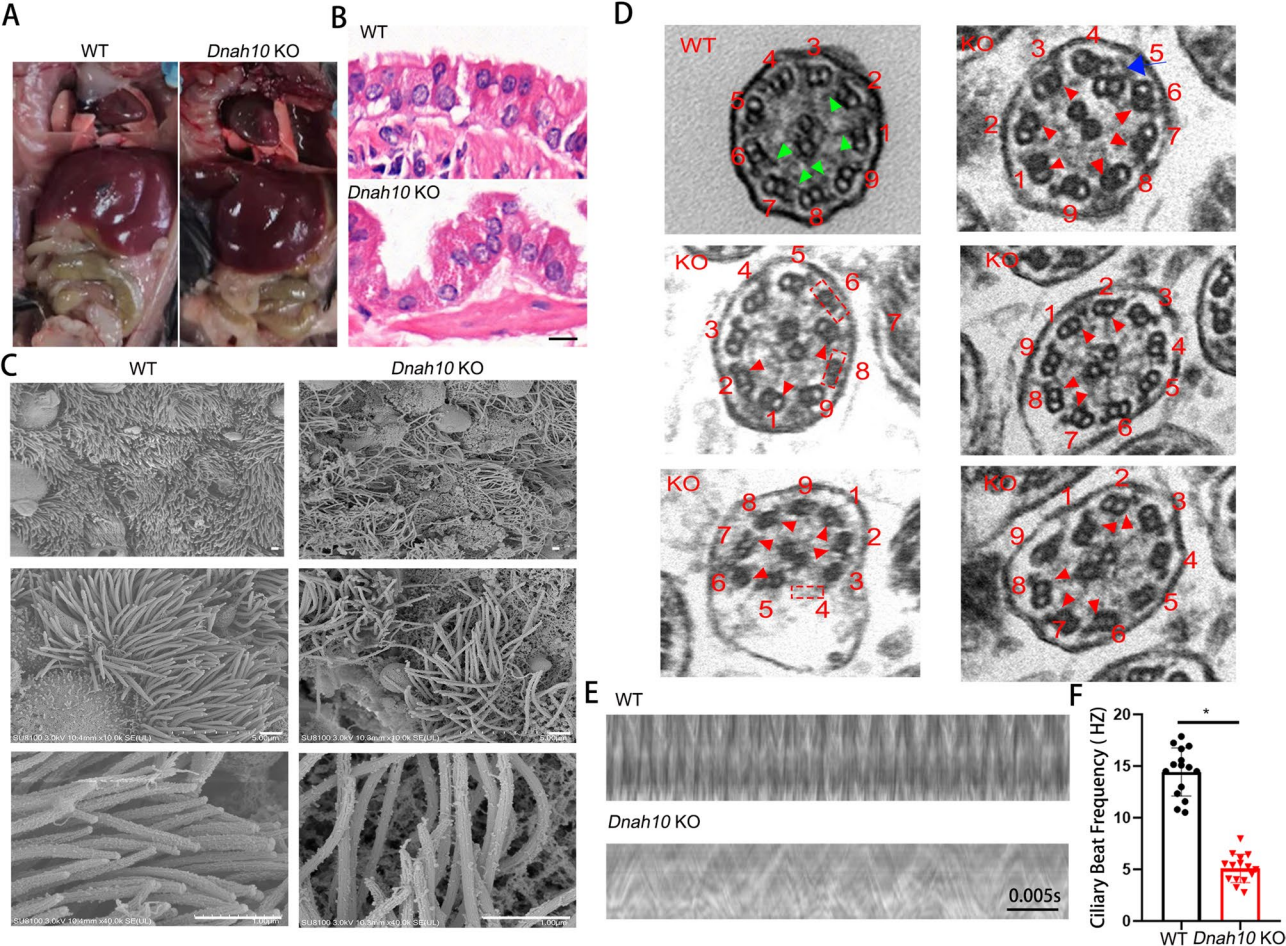
*Dnah10* KO mice showed typical PCD phototype. (**A**) Heterotaxy of the heart and intestines was observed in *Dnah10* KO mice. (**B**) H&E staining of bronchi in lung sections from 40-day-old WT and *Dnah10* KO mice revealed that, compared to WT mice, the tracheal epithelia of *Dnah10* KO mice exhibited a more clustered distribution of cilia. Scale bar = 10 μm. (**C**) The SEM images depicted the surface of tracheal epithelia from both WT and *Dnah10* KO mice. Scale bar = 9 μm. (**D**) TEM images of tracheal epithelium cilia were obtained from both WT and *Dnah10* KO mice. Compared to WT mice showing normal “9 + 2” axonemal structure, and intact IDAs (Green arrows), KO mice exhibited disruption of some ciliary axoneme structures, showing single microtubules, loss of a set of doublet microtubules (red dashed box), and absence of IDAs (the red arrow). Scale bar = 200 nm. (**E**) A representative kymograph of nasal cilia from 40-day-old WT and *Dnah10* KO mice was obtained through high-speed video microscopy. Scale bar = 0.005s. (**F**) The ciliary beat frequency (CBF) of *Dnah10* KO mice was significantly reduced compared to that of WT mice (*P < 0.05). Each group consisted of six mice

Notably, the *Dnah10* KO mice exhibited heterotaxy syndrome. The heart was located on the right side of *Dnah10* KO mice, whereas the other organs appeared to be appropriately distributed (Fig. 4A). This suggested that the defect in DNAH10 disrupts the node cilia. To investigate the phenotypic alterations in the airway motile cilia, we conducted H&E staining. Our findings revealed severe vacuolation in the multiciliated airway epithelial structure of *Dnah10* KO mice, accompanied by a more crooked appearance of the cilia (Fig. 4B). SEM was employed to visualize the morphology of the cilia. In contrast to the straight and uniformly directed cilia protruding from the cell plasma membrane in WT mice, the cilia in *Dnah10* KO mice displayed curvature, lacked directional orientation, and aberrantly protruded outside the cells and into the disrupted respiratory epithelium (Fig. 4C). Furthermore, the distal regions of the motile cilia in *Dnah10* KO mice displayed marked curvature (Fig. 4C). Additionally, TEM analysis revealed the absence of the IDA ultrastructure and disorganization of the outer doublet microtubules in the cilia region of the KO mice (Fig. 4D). Furthermore, high-speed video microscopy demonstrated a significant reduction in ciliary beat frequency (CBF) in the KO mice compared to the WT mice, suggestive of a phenotypic recapitulation of patients with PCD, thus confirming the impairment of ciliary motility due to DNAH10 deletion (Fig. 4E, F; Supplementary movie 1–2).

### DNAH10 defect impaired IDAf complex

**Fig. 5:**
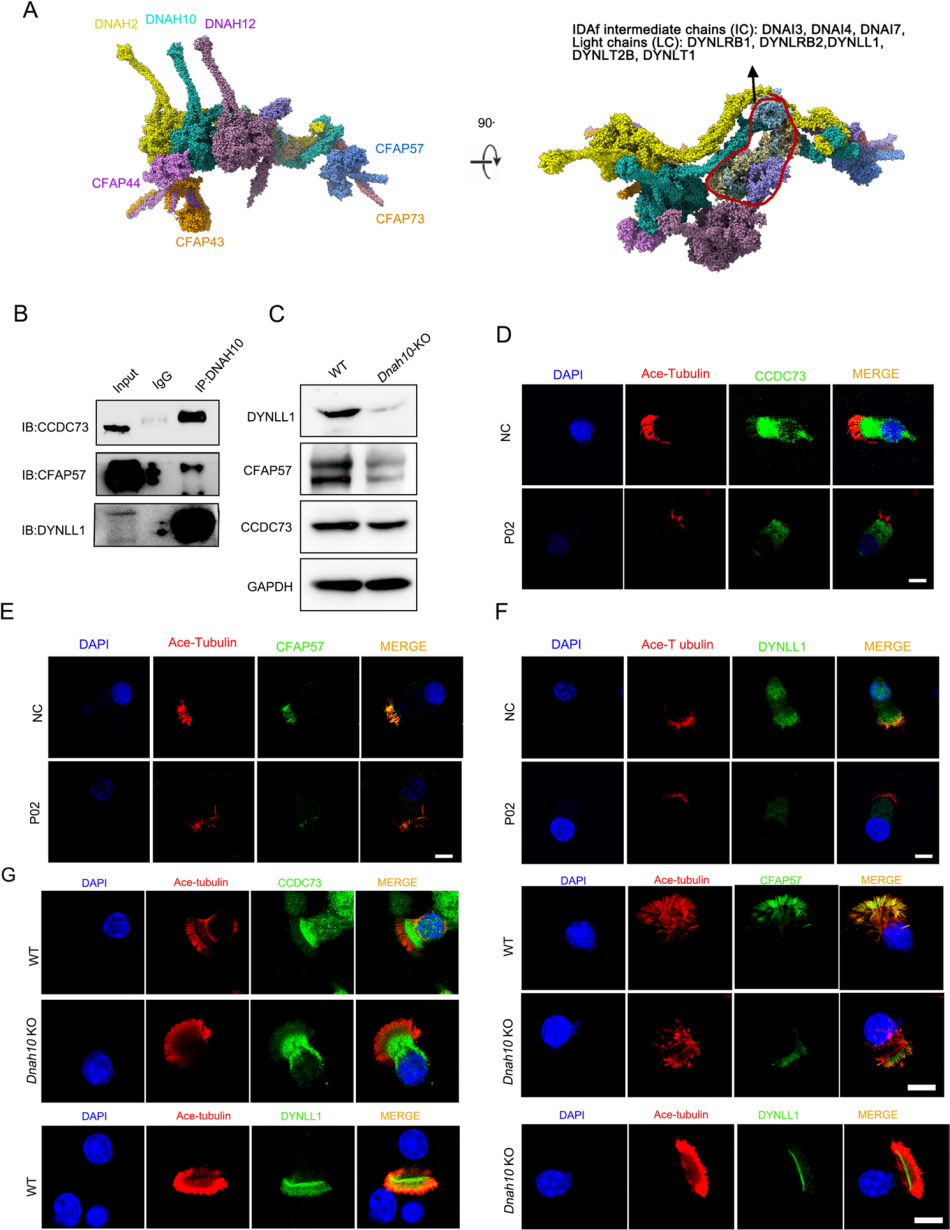
Cryo-electron microscopy identified interactors of DNAH10 in ciliated cells from *Dnah10* KO mice. (**A**) DNAH10 interacts with DNAH2 (Uniprot ID: Q9P225), DNAI3 (Q8IWG1), DNAI4 (Q5VTH9), DNAI7 (Q6TDU7), DYNLRB1 (Q9NP97), DYNLRB2 (Q8TF09), DYNLL1 (P63167), DYNLT2B (Q8WW35), DYNLT1 (P63172), CFAP43 (Q8NDM7), CFAP44 (Q96MT7), and DNAH12 (E9PG32) to form the IDAf complex using cryo-electron microscopy. (**B**) Co-IP analysis confirmed the interaction between DNAH10 and CCDC73/CFAP57/DYNLL1 in mouse lung tissues. (**C**) Western blot analysis was performed to assess the expression of CCDC73/CFAP57/DYNLL1, which was diminished in *Dnah10* KO mice. D-F. Immunofluorescence analysis revealed the expression of CCDC73/CFAP57/DYNLL1 was reduced in ciliated cells from the nasal mucous membrane of patient P02. Scale bars = 5 mm. Red, Ace-tubulin; Green, CCDC73, CFAP57, DYNLL1, respectively; blue, DAPI. G. Immunostaining analysis was performed to investigate the expression of CCDC73, CFAP57, and DYNLL1 in ciliated cells derived from *Dnah10* KO nasal epithelium. Red, ace-tubulin; green, CCDC73, CFAP57, and DYNLL1; blue, DAPI; Scale bar = 10 μm

**Fig. 6:**
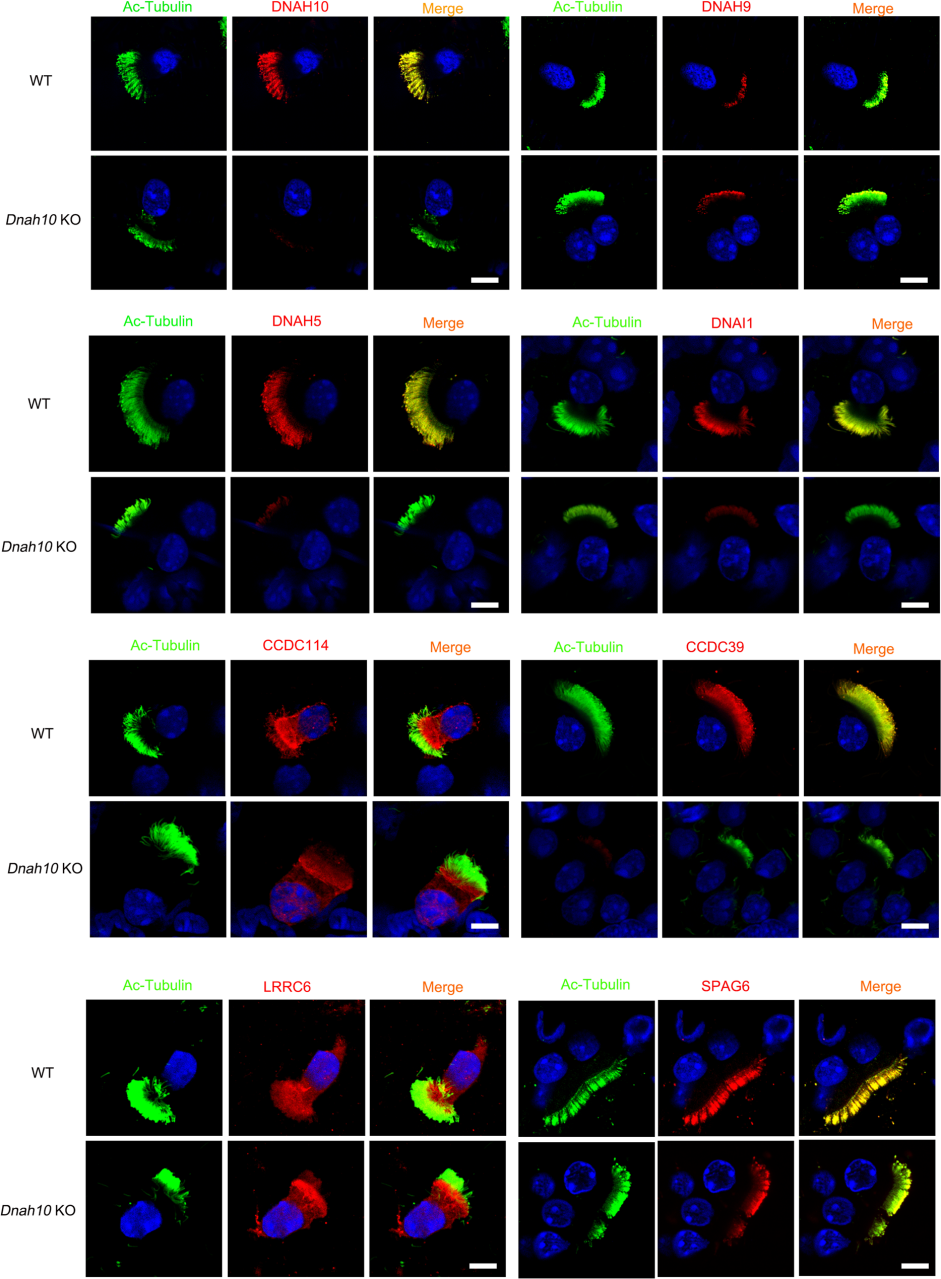
Immunostaining analysis was performed to investigate the expression of DNAH10, DNAH9, DNAH5, DNAI1, CCDC114, CCDC39, LRRC6, and SPAG6 in ciliated cells derived from *Dnah10* KO nasal epithelium. Green, ace-tubulin; red, DNAH10, DNAH9, DNAH5, DNAI1, CCDC114, CCDC39, LRRC6, and SPAG6; blue, DAPI; Scale bar = 10 μm

To investigate the role of DNAH10 in cilia motility, we utilized ciliary structure data obtained through cryogenic electron microscopy (cryo-EM) to gain structural insights into the IDAf complex. The IDAf complex comprises intermediate chains (DNAI3, DNAI4, DNAI7), light chains (DYNLRB1, DYNRB2, DYNLL1), and heavy chains (DNAH2, DNAH10, DNAH12). The spatial organization of the IDAf complex involves the assembly of DNAH2, DNAI3, DNAI4, DNAI7, DYNLRB1, DYNLRB2, DYNLL1, DYNLT2B, DYNLT1, CFAP43, CFAP44, CFAP57, CFAP73, CFAP100, and DNAH12 (Fig. 5A). To validate the structural information, we selected three proteins (CCDC73, CFAP57, DYNLL1) and conducted co-immunoprecipitation experiments using lung tissues. The results confirmed the interactions between DNAH10 and CCDC73, CFAP57, and DYNLL1, respectively (Fig. 5B). Western blot analysis revealed decreased expression levels of these proteins (Fig. 5C). Immunofluorescence staining revealed localization of CCDC73 and DYNLL1 in both cilia and cytoplasm, whereas CFAP57 was exclusively localized to the cilia. The expression levels of these proteins were found to be reduced in the cilia of knockout mice (Fig. 5D-F)and patient (Fig. 5G). To delve deeper into the potential impact of DNAH10 absence on proteins neighboring the IDAf, immunostaining was conducted to meticulously assess markers associated with the ODA (DNAH5, DNAH9), IDA light chain (DNAI1), dynein arm assembly (LRRC6), inner dynein arm assembly (CCDC39), outer dynein arm assembly (CCDC114), and the axoneme central apparatus protein SPAG6 in multiciliated cells derived from KO mice nasal tissue. Notably, our immunostaining results revealed a significant reduction in the expression of DNAH10, DNAH5, DNAI1, and CCDC39 in cilia upon deletion of DNAH10. In contrast, DNAH9, CCDC114, LRRC6, and SPAG6 were not significantly affected (Fig. 6). This finding suggested a structural alteration in the IDA-associated axonemal structure of *Dnah10* KO mice.

### *Danh10* KO mice presented with mitochondrial metabolic rewiring and pulmonary fibrosis

**Fig. 7:**
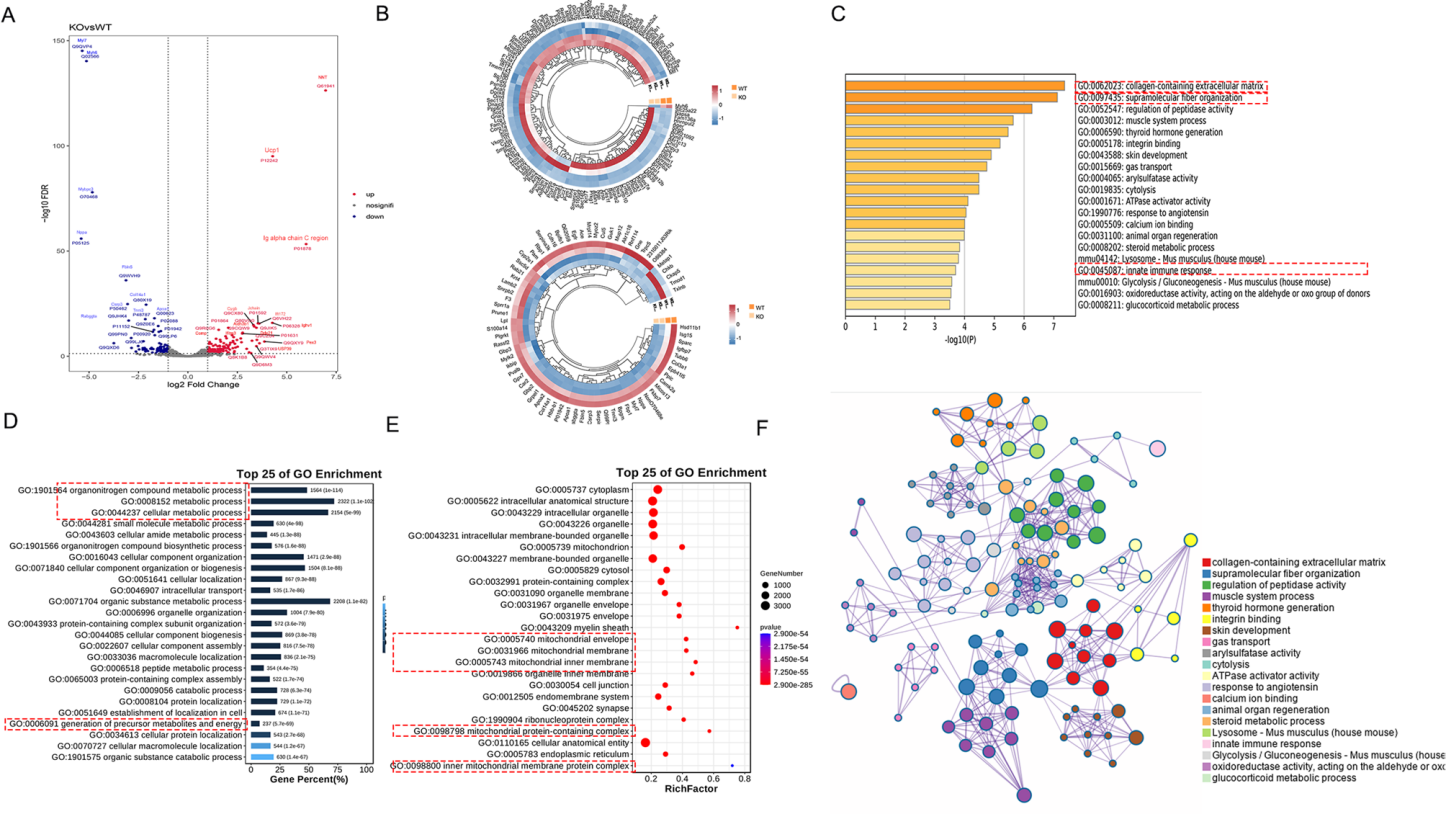
LC-MS/MS proteomics identified upregulated and downregulated proteins in lung tissues from *Dnah10*KO *mice.* (**A**) Volcano plot depicted protein alterations in lung tissues of WT and *Dnah10* KO mice. (**B**) The circular heatmap illustrates differential gene expression patterns between WT and *Dnah10* KO mice. C, D. GO pathway analysis revealed enrichment of 147 significantly downregulated and 916 upregulated proteins, based on functional annotation from OmicShare. The upregulated proteins were primarily associated with collagen-rich extracellular matrix, supramolecular fiber organization, and immune response, whereas the downregulated proteins were linked to metabolic processes. E. The Gene Ontology (GO) Cellular Component Ontology described many decreased proteins belong to mitochondrial components. F. The correlation between down-regulated proteins from GO pathway analysis was analyzed

To further investigate PCD pathophysiology caused by deletion of DNAH10, we employed a proteomics approach for WT mice and *Dnah10* KO. A total of 4570 proteins were quantified, revealing 349 upregulated proteins and 90 downregulated proteins (Fig. 7A, B, Supplementary Fig. 4). Heat map donut charts provided detailed visualizations of the upregulated and downregulated proteins influenced by the deletion of *Dnah10* (Fig. 7B). GO enrichment analysis demonstrated significant alterations in gene categories in response to the deletion of *Dnah10*. The upregulated GO terms suggested a significant enrichment of collagen-containing extracellular matrix, super-molecular fiber organization, and innate immune response pathway in *Dnah10* KO mice (Fig. 7C). The downregulated proteins were associated with metabolic processes, cellular component assembly, and macromolecule localization (Fig. 7D). We observed that some of the downregulated proteins belonged to mitochondria (Fig. 7E). Furthermore, a network diagram was utilized to analyze the interactions of differential proteins, known to play crucial roles in fibrosis in humans and/or mice, between *Dnah10* KO mice and WT mice. The results revealed complex interactions among proteins involved in these processes (Fig. 7F).

**Fig. 8:**
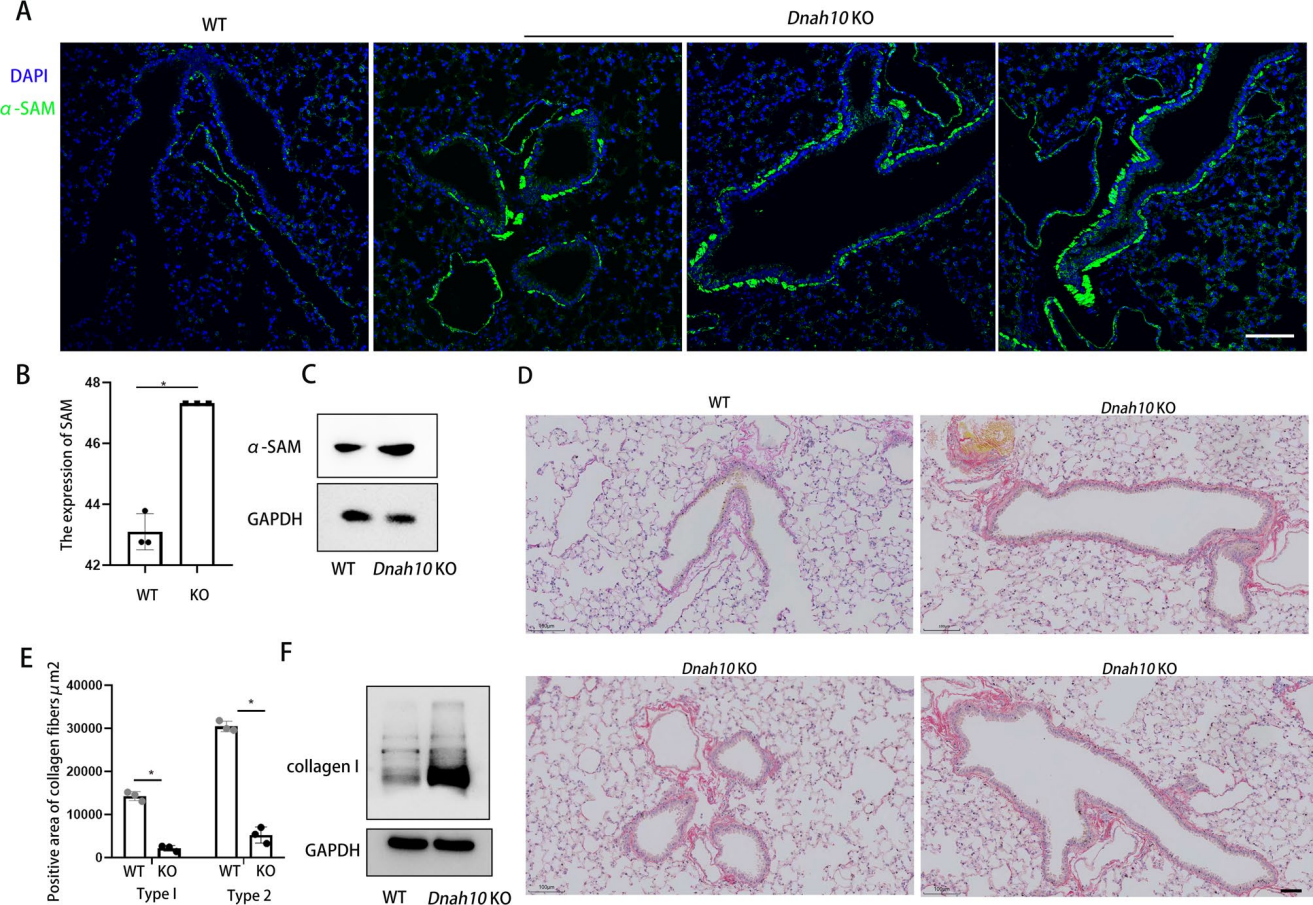
*Dnah10* KO mice exhibited pulmonary fibrosis phenotype. (**A**) Immunofluorescence revealed a significant increase in α-SMA staining in *Dnah10* KO mice. α-smooth muscle actin (α-SMA) is a biomarker for the assessment of activated fibroblasts. Scale bar = 50 μm. Green, α-SMA; Blue, DAPI. (**B**) Results of immunofluorescence images quantification corresponding to α-SMA in (**A**). (**C**) Western blot analysis confirmed the increased expression of α-SMA. D, E. Sirius red staining demonstrated a notable elevation in both type I and type II collagen fibers in *Dnah10* KO mice. Scale bar = 50 μm. F. Western blot analysis confirmed the increased expression of collagen I

Subsequently, we investigated the pulmonary fibrosis phenotype in *Dnah10* KO mice. ECM remodeling serves as a pivotal process in pathologies, including fibrosis [18, 19]. Fibroblasts represent the predominant cell type in connective tissues throughout the body and are the primary source of ECM components [20]. Fibroblasts primarily function in maintaining and synthesizing new fibrillar collagens, thereby preserving tissue homeostasis. Upon activation, fibroblasts undergo transdifferentiation into myofibroblasts, triggered by chemical signals promoting either proliferation or cellular differentiation. This process leads to excessive collagen deposition and tissue remodeling. Consequently, myofibroblasts are implicated in the increased stiffness of the ECM, a characteristic observed in fibroproliferative diseases. Myofibroblasts uniformly express α-smooth muscle actin (α-SMA), a 42 kDa actin isoform typically found in stem and precursor cells [21]. α-SMA serves as a well-established biomarker for the assessment of activated fibroblasts across various tissues and organs, including the lung. Immunofluorescence and Western blot revealed a significant increase in α-SMA staining in *Dnah10* KO mice (Fig. 8A-C). Additionally, Sirius red staining demonstrated a notable elevation in both type I and type II collagen fibers in *Dnah10* KO mice (Fig. 8D, E). Western blot also demonstrated elevated expression of collagen I protein (Fig. 8F). Collectively, our findings demonstrated that DNAH10 deletion led to pulmonary fibrosis in *Dnah10* KO mice.

## Discussion

In this study, we identified biallelic *DNAH10* variants in two individuals with PCD and observed that the *Dnah10* KO mouse model recapitulated the PCD features observed in these two subjects. Functionally, DNAH10 can cooperated with CCDC73, CFAP57 and DYNLL1 to form IDAf complex. Defects in DNAH10 led to an aberrant immune response, pulmonary fibrosis, and disrupted mitochondrial metabolic processes.

The inner dynein arms (IDAs) and outer dynein arms (ODAs) are essential components of motile cilia, responsible for hydrolyzing adenosine triphosphate (ATP) to generate ciliary beating power. They also modulate beat frequency and pattern through their interactions with the axonemal complex. Consequently, the DNAH family of genes plays a pivotal role in causing complex phenotypes associated with motile ciliopathies. DNAH5, DNAH9, and DNAH11, members of the DNAH family that encode ODA components in motile cilia, have been implicated in PCD [15, 22, 23]. DNAH6 is an IDA protein component, and defects in it can lead to heterotaxy and primary ciliary dyskinesia [24]. Only one study has reported that mutations in *DNAH1*, which encodes IDA components, are associated with PCD [25]. Our study provided new evidence to confirm that *DNAH10* is a pathogenic gene for PCD in human. First, we identified two individual who carried *DNAH10* mutations were diagnosed as PCD. Second, we verified the expression of DNAH10 was decreased in patients. Third, *Dnah10* KO mouse showed similar phenotypes, including abnormal cilial morphology, slower cilia wave, compromised lung function, pulmonary inflammation and laterality defects.

Although a previous study identified a mutation in *DNAH10* in a patient with PCD, the pathogenesis of DNAH10 in ciliogenesis remained unexplored [17]. We utilized structural information from cryo-electron microscopy to identify components of the IDAf complex, including DNAH2, DNAI3, DNAI4, DNAI7, DYNLRB1, DYNLRB2, DYNLL1, DYNLT2B, DYNLT1, CFAP43, CFAP44, CFAP57, CFAP73, CFAP100, and DNAH12. These findings can provide valuable insights into potential PCD candidate genes. Additionally, we demonstrated that DNAH10 interacts with CCDC73, CFAP57, and DYNLL1 to regulate ciliary movement. DNAH10 deficiency led to obviously diminished expression of DYNLL1. DYNLL1 downregulation significantly impacted immune activation by regulating NF-κB subunits, as shown in a previous study [26]. In addition, we also found lack of DNAH10 disrupted the stability of DNAH5, DNAI1, CCDC114 and CCDC39. In summary, disruption of DNAH10 would render the IDAf complex instability, altering the beating frequency and direction of motile cilia.

One of novel finding in the current study is that loss of DNAH10 function led to upregulation of key proteins associated with the fibrosis and collagen-rich extracellular matrix and immune response. Additionally, airway-centered interstitial fibrosis (ACIF) was observed in *Dnah10* KO mice. ACIF is characterized by fibrotic changes in the respiratory bronchioles and the surrounding peribronchiolar interstitium. *Dnah10* KO mice presented an accumulation of extracellular matrix (ECM) in the airway produced by myofibroblasts expressing smooth muscle cell markers such as α-SMA. A recent finding has shown that cilia gene can directly involve in the liver fibrosis through in Hematopoietic stem cells (HSC cell) [27]. Whether DNAH10 is expressed in the primary cilia and regulate the primary cilia function need to investigate further. Furthermore, our findings revealed that the absence of DNAH10 led to abnormal mitochondrial metabolic processes, which piqued our interest. In a separate study, we discovered that DNAH10 regulated PACRG, a protein crucial for mitochondrial function and involved in NF-κB signaling to trigger apoptosis [28]. The precise mechanism underlying how the absence of DNAH10 impaired lung metabolic processes remained to be further elucidated.

Therapeutic strategies for PCD primarily aim to alleviate specific symptoms and complications exhibited by patients. The management of PCD typically necessitates a multidisciplinary approach, encompassing respiratory care, nutritional support, and surgical interventions. Respiratory care primarily focuses on maintaining airway clearance and preventing respiratory infections. Nutritional support aims to ensure adequate caloric intake and maintain nutritional balance. Surgical intervention may be necessary in severe cases to address anatomical abnormalities or enhance lung function [29]. Nevertheless, the effectiveness of these therapeutic strategies remains limited owing to the complexity and heterogeneity inherent to PCD. For PCD management, the prescription of suitable medications aimed at restoring cilia function is paramount. But now, there is currently no available treatment for defective airway cilia, the minute hair-like projections lining the respiratory tract. Our proteomics data further revealed that deficiencies in DNAH10 elicited abnormalities in proteins associated with mitochondrial metabolism, implying that impairments in cilium-related proteins, including DNAH9 and DNAH10, may contribute to mitochondrial metabolic disorders. The mechanism of mutual regulation between cilia and mitochondria remains unclear. A recent study reported a mechanistic link between mitochondria metabolism and primary cilia signaling. The study revealed that the loss of NDUFAF2 results in both mitochondrial and ciliary defects, both in vitro and in vivo [30]. Whether regulating mitochondrial metabolism can partially restore cilium function remains to be further investigated. NADH and ATP play a role in restoring abnormal ciliary beat patterns resulting from mutations in *DNAH9*, which encodes the heavy chain proteins of ODA [31]. NADH and ATP may represent promising therapeutic candidates for the treatment of PCD induced by dyneins defects.

In conclusion, we identified two individuals harboring bi-allelic variants of *DNAH10*. Genetic evidence derived from patients with *DNAH10*-associated conditions and knockout mice lacking *Dnah10* strongly supported the conclusion that bi-allelic *DNAH10* variants can trigger PCD. As a new finding, our research further elucidated the constitute of the IDAf complex containing DNAH10. Loss of DNAH10 function led to disorganized IDAf complex, accumulation of extracellular matrix (ECM) and abnormal mitochondrial metabolism. In conclusion, our study demonstrated a mechanistic relationship between cilia and mitochondrial metabolism, offering a therapeutic perspective for patients with ciliopathies.

## Supplementary Information

Below is the link to the electronic supplementary material.

Supplementary Material 1

Supplementary Material 2

Supplementary Material 3

Supplementary Material 4

Supplementary Material 5

Supplementary Material 6

Supplementary Material 7

Supplementary Material 8

Supplementary Material 9

Supplementary Material 10

Supplementary Material 11

## Data Availability

All relevant data are in the figures and supplementary figures. Any raw data and materials that can be shared will be released via a material transfer agreement. Proteomic data could be made available upon requests directed to the corresponding author.
