# Supplementary figures and images for: DNAH10 mutation cause primary ciliary dyskinesia with defects of IDAf complex assembly and lung fibrosis manifestation
Source: Orphanet J Rare Dis. 2025 Sep 2;20:469. doi: 10.1186/s13023-025-03977-w (PMC12403265; doi:10.1186/s13023-025-03977-w)

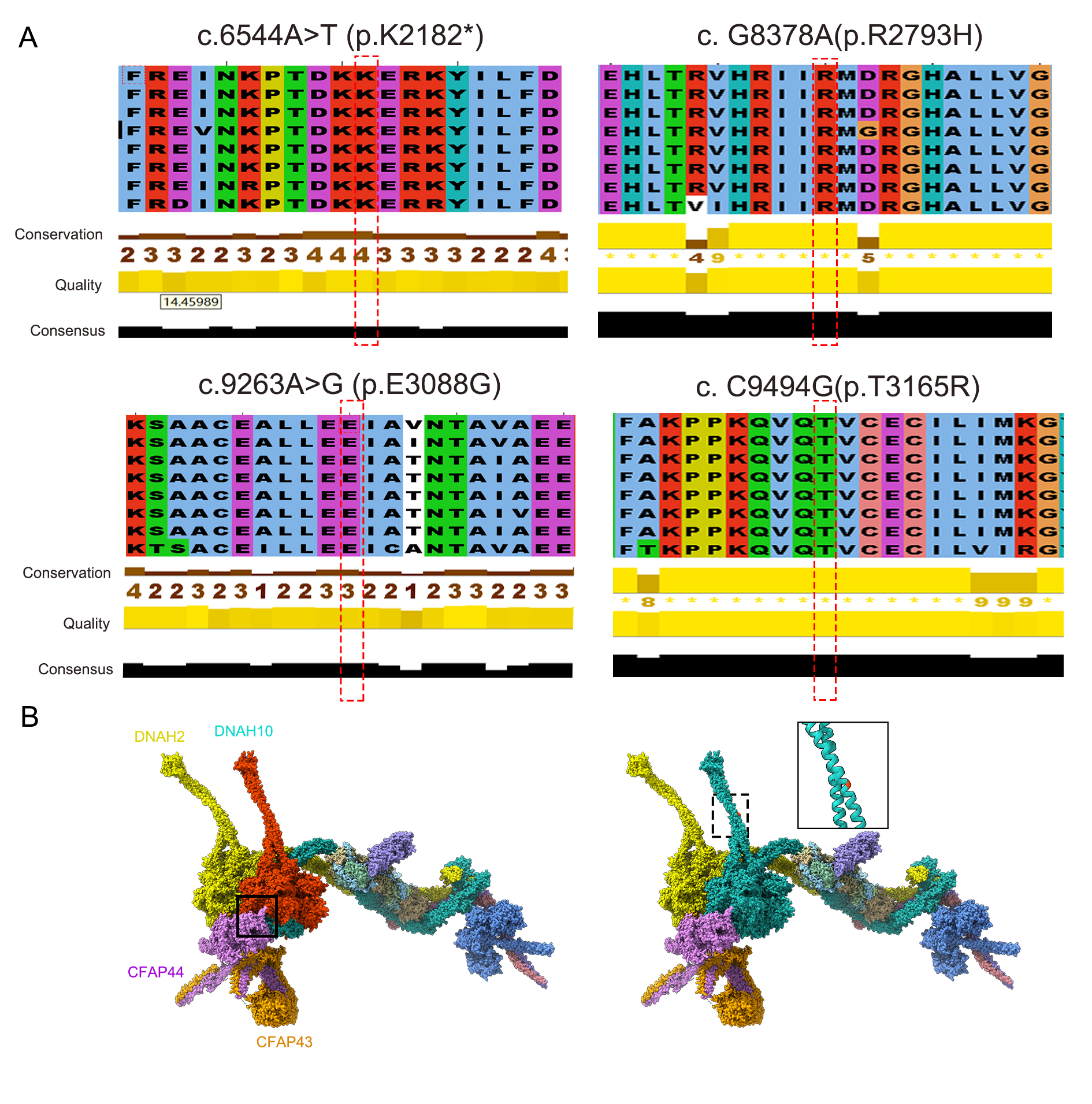

Supplement: Supplementary file 1 — Supplementary Material 1 [file 13023_2025_3977_MOESM1_ESM.png]

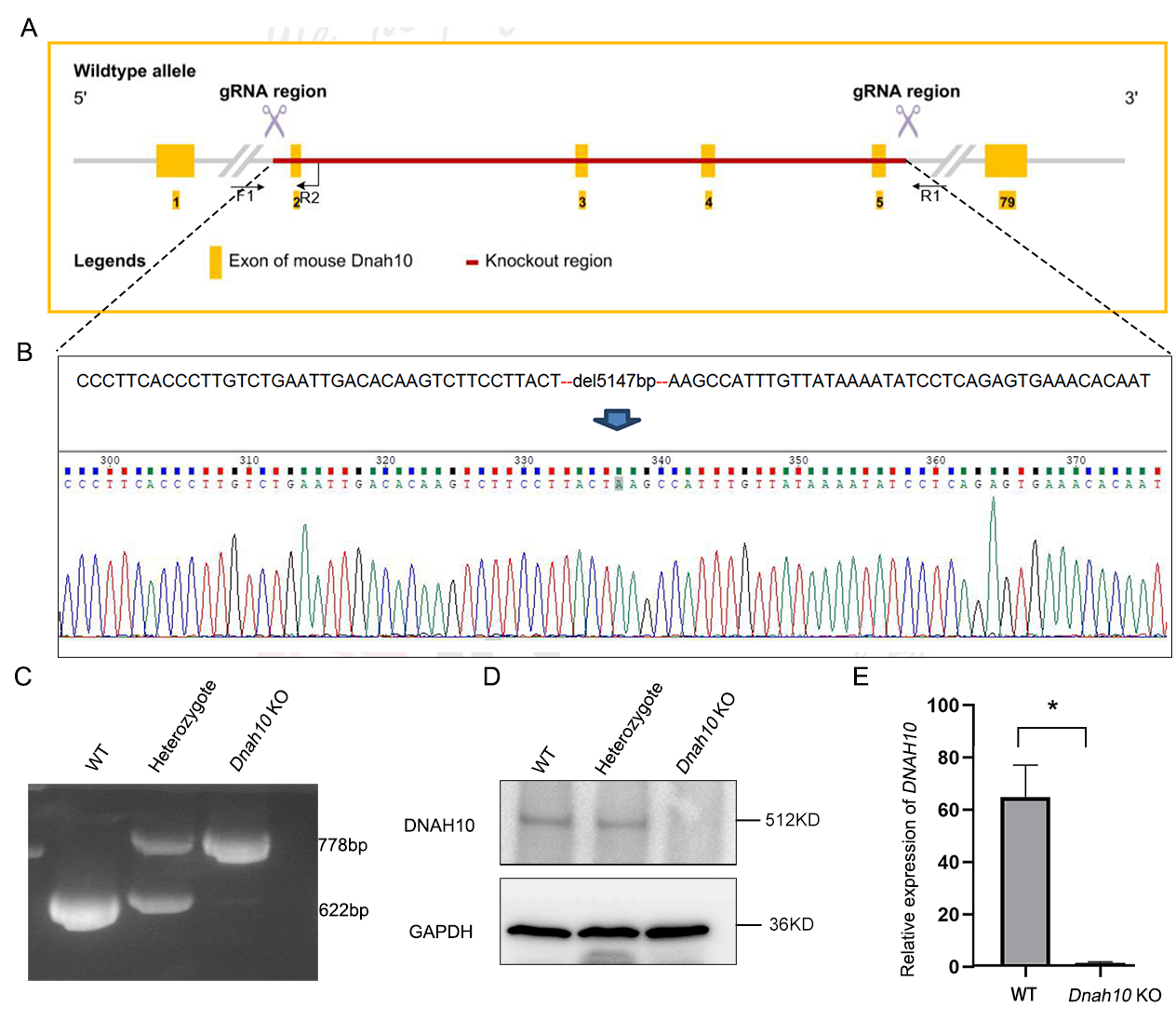

Supplement: Supplementary file 2 — Supplementary Material 2 [file 13023_2025_3977_MOESM2_ESM.png]

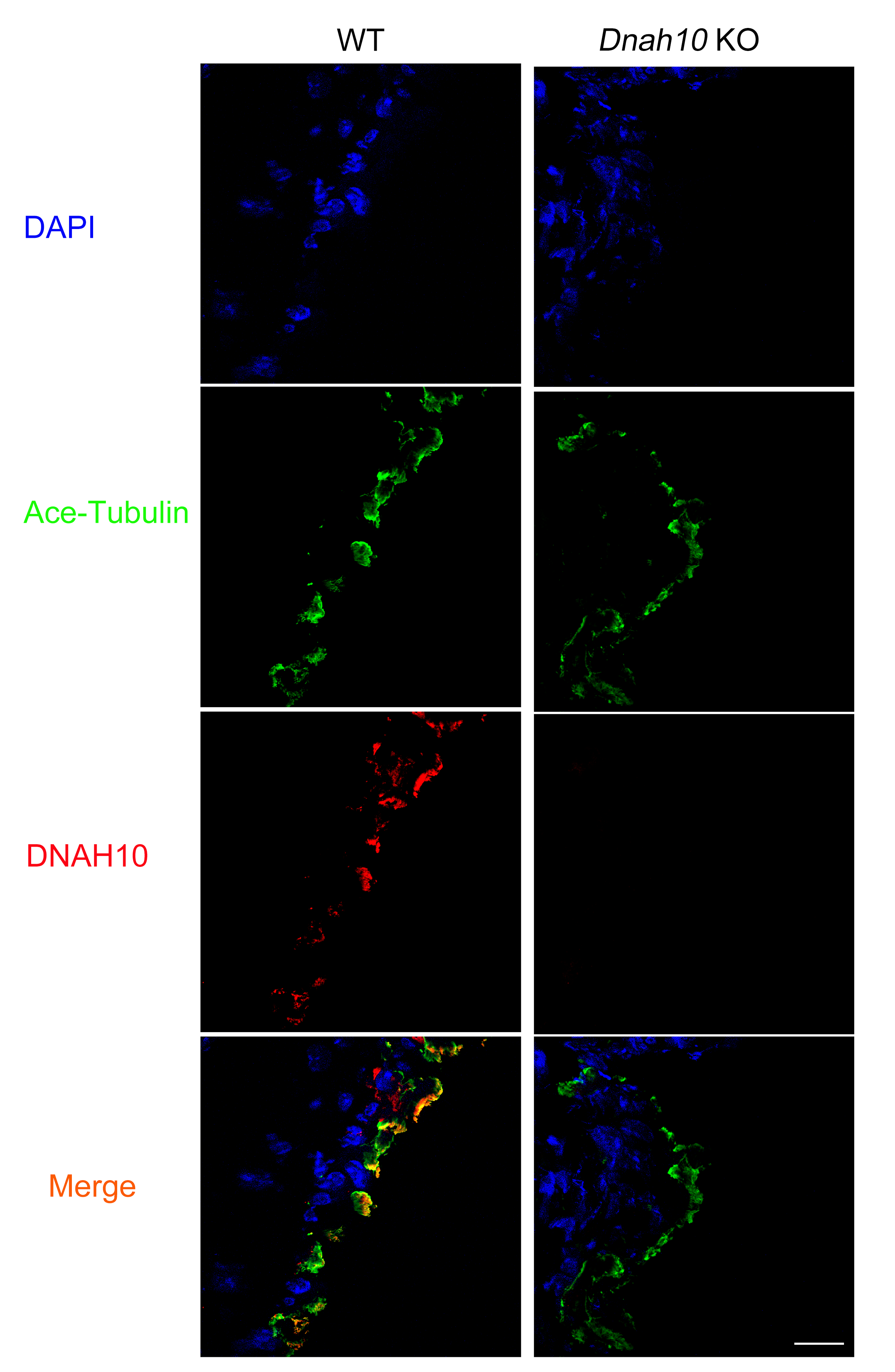

Supplement: Supplementary file 3 — Supplementary Material 3 [file 13023_2025_3977_MOESM3_ESM.png]

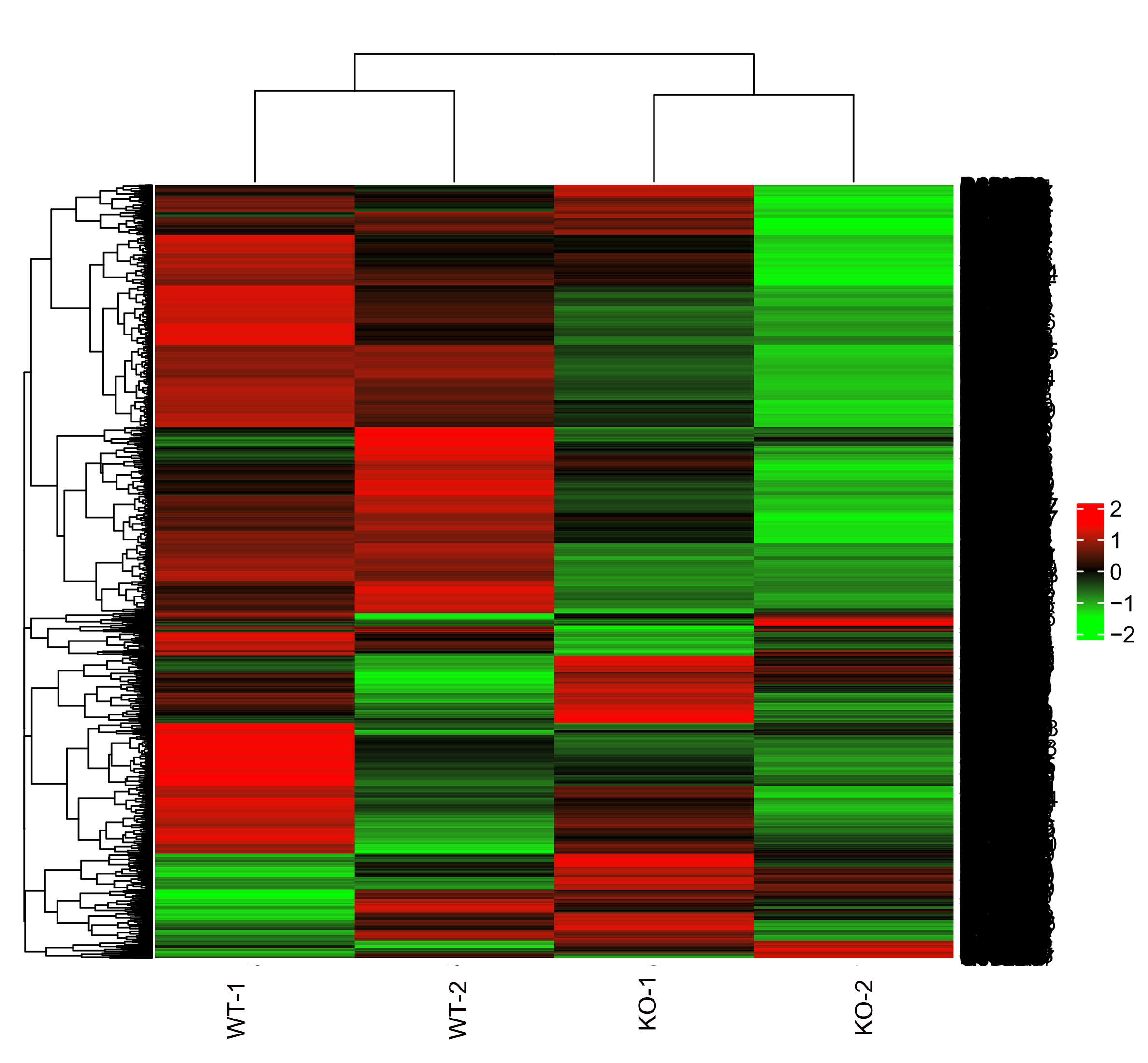

Supplement: Supplementary file 4 — Supplementary Material 4 [file 13023_2025_3977_MOESM4_ESM.png]
