# Supplementary material for: DNAH10 mutation cause primary ciliary dyskinesia with defects of IDAf complex assembly and lung fibrosis manifestation
Source: Orphanet J Rare Dis. 2025 Sep 2;20:469. doi: 10.1186/s13023-025-03977-w (PMC12403265; doi:10.1186/s13023-025-03977-w)
Supplement: Supplementary file 5 — Supplementary Material 5 [file 13023_2025_3977_MOESM5_ESM.docx]

**Supplementary Table S1 Overview of the information of primer sequences in this paper**

| Application of primers | Sequences |
| --- | --- |
| Sanger sequencing primer of *DNAH10* (c.A6544T) | F 5' -gggctgacgacaaagttgtac-3'  R 5' -atgtatagtcctgccaccg-3' |
| Sanger sequencing primer of *DNAH10* (c.A9263G) | F 5' -ggccacctcgacgatgaac-3'  R 5' -gtattgggtgttggggatgggc-3' |
| Sanger sequencing primer of *DNAH10* (c.G8378A) | F 5' - cacatcctctccagcacctg-3'  R 5' -accaactgcagaaactggca-3' |
| Sanger sequencing primer of *DNAH10* (c.C9494G) | F 5' -caggagtgcggtttgtgttg-3'  R 5' -actagtgccgaatcgaaccc-3' |
| gRNA-1 sequence | F 5' -ATCTTATGCTGGGTCCAGTAAGG,-3' |
| gRNA-2 sequence | R 5' - TTTATAACAAATGGCTTGATTGG-3' |
| *Dnah10* KO genotyping (F/R1) | F 5' -GGTGCCTTCACTCTGTAAGTGTCTTGGTCTC-3'  R 5' -GCTTCATTTCCTCCAGACACCCACCTG-3' |
| *Dnah10* KO genotyping (F/R2) | F 5' -GGTGCCTTCACTCTGTAAGTGTCTTGGTCTC-3'  R 5' -GCTGTCTGTTACACTGATGGATGTGCAAGTCG-3' |
| RT-PCR primer of *Dnah10* | F 5' -AGCGATCATAGACAGGGGGA-3'  R 5' -CGTTCAGCGTGTTGATGAGC-3' |
