# Supplementary material for: DNAH10 mutation cause primary ciliary dyskinesia with defects of IDAf complex assembly and lung fibrosis manifestation
Source: Orphanet J Rare Dis. 2025 Sep 2;20:469. doi: 10.1186/s13023-025-03977-w (PMC12403265; doi:10.1186/s13023-025-03977-w)
Supplement: Supplementary file 6 — Supplementary Material 6 [file 13023_2025_3977_MOESM6_ESM.docx]

**Supplementary Table S2 Overview of the antibodies or dye information in this study**

| Antibody or dye (application and dilution ratio) | Source | Identifier |
| --- | --- | --- |
| Anti-acetyl-α-Tubulin (IF: 1:200) | CST | D20G3 |
| Anti-DNAH10 (WB: 1:500; IF: 1:100) | Proteinatlas | HPA039066 |
| Anti-GAPDH (WB: 1:1000) | Proteintech | 60004-1-Ig |
| Anti-IFN-β (WB: 1:1000) | Abcam | ab85803 |
| Anti-CD68 (WB: 1:500; IF: 1:200) | Abcam | ab125212 |
| Anti-CD45 (WB: 1:500; IF: 1:200) | Abcam | ab10558 |
| Anti-CD63 (WB: 1:500; IF: 1:100) | Proteintech | 25682-1-AP |
| Anti-CD86 (WB: 1:500; IF: 1:100) | Proteintech | 26903-1-AP |
| Anti-CCDC37 (WB: 1:500; IF: 1:50) | Biobyt | orb2330 |
| Anti-CFAP57 (WB: 1:500; IF: 1:100) | Biobyt | orb30169 |
| Anti-DYNLL1 (WB: 1:500; IF: 1:100) | Proteintech | 18130-1-AP |
| Rabbit IgG control (IP: 1:100) | Proteintech | 30000-0-AP |
| Mouse Control IgG (IP: 1:100) | ABclonal | AC011 |
| Anti-α-SMA (IF: 1:200) | Abcam | ab5694 |
| Anti-p63 (IF: 1:200) | Abcam | ab124762 |
| Anti-KRT5 (IF: 1:200) | Abcam | ab52635 |
| Anti-Muc5AC (IF: 1:200） | Abcam | ab3649 |
| Anti-CC-10 (IF: 1:50) | Santa Cruz Biotechnology | sc-365992 |
| Anti-DNAH9 (IF: 1:50) | Thermo Fisher | PA5-45744 |
| Anti-DNAH5 (IF: 1:200) | Abcam | ab234826 |
| Anti-DNAI1 (IF: 1:100) | Proteintech | 12756-1-AP |
| Anti-CCDC14 (IF: 1:100) | Proteintech | 27211-1-AP |
| Anti-CCDC39 (IF: 1;100) | Proteintech | 29817-1-AP |
| Anti-LRRC6 (IF: 1;100) | Origene | OTI7E9 |
| Anti-SPAG6 (IF: 1:100) | Proteintech | 12462-1-AP |
| Goat Anti-Rabbit IgG (H + L)-HRP Conjugate（WB: 1:3000） | Bio-Rad | 1706515 |
| Goat Anti-Mouse IgG (H + L)-HRP Conjugate（WB: 1:3000） | Bio-Rad | 1706516 |
| Goat anti-Rabbit IgG (H+L), Alexa Fluor™ 488 (IF: 1:1000) | Thermo Fisher | A-11008 |
| Goat anti-Rabbit IgG (H+L), Alexa Fluor™ 594 (IF: 1:1000) | Thermo Fisher | A-11012 |
| Goat anti-Mouse IgG (H+L), Alexa Fluor™ 488 (IF: 1:1000) | Thermo Fisher | A-11001 |
| Goat anti-Mouse IgG (H+L), Alexa Fluor™ 594 (IF: 1:1000) | Thermo Fisher | A-11005 |
