# Supplementary material for: DNAH10 mutation cause primary ciliary dyskinesia with defects of IDAf complex assembly and lung fibrosis manifestation
Source: Orphanet J Rare Dis. 2025 Sep 2;20:469. doi: 10.1186/s13023-025-03977-w (PMC12403265; doi:10.1186/s13023-025-03977-w)
Supplement: Supplementary file 7 — Supplementary Material 7 [file 13023_2025_3977_MOESM7_ESM.docx]

**Supplemental Table S3 variant analysis of patient harboring *DNAH10* mutations**

| Genomic mutation | Protein changes | Mutation type | Genotype | Allele frequency in ExAC | 1000 Genomes Project | GnomAD | SIFT | Proven prediction | Mutation Taster |
| --- | --- | --- | --- | --- | --- | --- | --- | --- | --- |
| c.6544A>T | p. K2182* | nonsense | Heterozygous | 0 | 0 | 0 | D | D | D |
| c.9263A>G | p. E3088G | Missense | Heterozygous | 0 | 0 | 0 | D | D | D |
| c.9494C>G | p. T3165R | Missense | Heterozygous | 0.0001 | 0.0012 | 0.0001 | T | D | D |
| c.8378G>A | p. R2793H | Missense | Heterozygous | 0.0005 | 0.0019 | 0.0004 | D | D | D |

a: D means deleterious, T means tolerate in SIFT, b: D means probably damaging in Proven prediction, c: D means disease causing in Mutation Taster.
